# Supplementary material for: Improvement of the memory function of a mutual repression network in a stochastic environment by negative autoregulation
Source: BMC Bioinformatics. 2019 Dec 27;20:734. doi: 10.1186/s12859-019-3315-2 (PMC6935196; doi:10.1186/s12859-019-3315-2)
Supplement: Supplementary file 1 — Additional file 1: Text S1. Parameter settings of the MRN-NA. Text S2. One-variable equation and noise function of the MRN-NA. Text S3. Stochastic potential profile analysis. We estimated the stochastic potential profile of the one-variable rate equation derived for the MRN and MRN-NA models as described in the Appendix F of reference [12]. Figure S1. Bifurcation analysis of the MRN model and MRN-NA models. Figure S2. Stochastic simulations for 2000 simulation steps. Figure S3. Stochastic simulation of the MRN for 20000 simulation time steps. Figure S4. Stochastic fluctuations after the signal period. Figure S5. Stochastic potential profile of the MRN and MRN-NA models. [file 12859_2019_3315_MOESM1_ESM.pdf]

Supplementary Material

Improvement of the memory function of a mutual repression network in a stochastic environment by negative autoregulation

A B M Shamim Ul Hasan[1,2], Hiroyuki Kurata[2,*], Sebastian Pechmann[1,*]

[1] Department of Biochemistry, Université de Montréal, 2900 Edouard Montpetit Blvd, Montreal, QC, H3T 1J4, Canada

[2] The Biomedical Informatics R&D Center, Kyushu Institute of Technology, 680-4 Kawazu, Iizuka, Fukuoka 820-8502, Japan

* E-mail: kurata@bio.kyutech.ac.jp (HK); sebastian.pechmann@umontreal.ca (SP)

Text S1

Parameter settings of the MRN-NA

The equations of the MRN-NA model are given by Eqs. (1-3). When the corresponding parameters between Eqs. (2, 3) are set to be the same as follows:

$$k(3)=k(6),\ k(5)=k(8),\ k(9)=k(10), K(1)=K(3),\ K(2)=K(4),\ K(5)=K(6)$$

the steady state solution is given by:

$$y_{ss}(1)=\frac{k(1).S}{k(2)} \tag{S1}$$

$$y_{ss}(2)=[\{k(6).\frac{y_{ss}(1)}{y_{ss}(1)+K(1)}+k(4).\frac{K(2)^n}{y_{ss}(3)^n+K(2)^n}-k(5).K(5)\}$$

$$+\{(k(6).\frac{y_{ss}(1)}{y_{ss}(1)+K(1)}+k(4).\frac{K(2)^n}{y_{ss}(3)^n+K(2)^n}-k(5).K(5))^2$$

$$+4.k(5).K(5).(k(9)+k(6).\frac{y_{ss}(1)}{y_{ss}(1)+K(1)}$$

$$+k(4).\frac{K(2)^n}{y_{ss}(3)^n+K(2)^n})\}^{1/2}].\frac{1}{2.k(5)}$$

(S2)

$$y_{ss}(3)=[\{k(6).\frac{K(1)}{y_{ss}(1)+K(1)}+k(7).\frac{K(2)^n}{y_{ss}(2)^n+K(2)^n}-k(5).K(5)\}$$

$$+\{(k(6).\frac{K(1)}{y_{ss}(1)+K(1)}+k(7).\frac{K(2)^n}{y_{ss}(2)^n+K(2)^n}-k(5).K(5))^2$$

$$+4.k(5).K(5).(k(9)+k(6).\frac{K(1)}{y_{ss}(1)+K(1)}$$

$$+k(7).\frac{K(2)^n}{y_{ss}(2)^n+K(2)^n})\}^{1/2}].\frac{1}{2.k(5)}$$

(S3)

where

$$k(4) = \{k(5).y_{ss}(2) - k(6).\frac{y_{ss}(1)}{y_{ss}(1)+K(1)}$$

$$- k(9).\frac{K(5)}{y_{ss}(2)+K(5)}\}.\frac{y_{ss}(3)^n + K(2)^n}{K(2)^n}$$

(S4)

$$k(7) = \{k(5).y_{ss}(3) - k(6).\frac{K(1)}{y_{ss}(1)+K(1)}$$

$$- k(9).\frac{K(5)}{y_{ss}(3)+K(5)}\}.\frac{y_{ss}(2)^n + K(2)^n}{K(2)^n}$$

(S5)

A value of $k(7)$ is fixed and a value of $k(4) > k(7)$ is determined so that the high levels of $y_{ss}(2)$ and $y_{ss}(3)$ can be conserved. The expression levels of $y_{ss}(2)$ and $y_{ss}(3)$ are always as similar as possible between the MRN and MRN-NA models.

Text S2

One-variable equation and noise function of the MRN-NA

To perform deterministic and stochastic double-well potential analyses, we converted the reaction rate equations (Eqs. (1-3)) into the corresponding one-variable rate equation [12].

From setting $S = 0$ at steady state in Eqs. (1-3), the MRN-NA model is given by

$$\frac{dy(2)}{dt} = k(4).\frac{K(2)^n}{y(3)^n + K(2)^n} + k(9).\frac{K(5)}{y(2)+K(5)} - k(5).y(2) \tag{S6}$$

$$\frac{dy(3)}{dt} = k(6) + k(7).\frac{K(4)^n}{y(2)^n + K(4)^n} + k(10).\frac{K(6)}{y(3)+K(6)} - k(8).y(3) \tag{S7}$$

We solved the probability density at the steady state (at $t \to \infty$). In the limit $t \to \infty$ $y(2)$ and $y(3)$ approach the steady state, i.e., $\frac{dy(2)}{dt} \to 0$ and $\frac{dy(3)}{dt} \to 0$. Therefore, we assumed $\frac{dy(3)}{dt} = 0$ to solve the ODE of $\frac{dy(2)}{dt}$ at $t \to \infty$. In a similar manner, we assumed $\frac{dy(2)}{dt} = 0$ to solve the ODE of $\frac{dy(3)}{dt}$ at $t \to \infty$.

By applying the quasi-steady-state approximation [12, 50, 53] to $y(3)$, the one-variable rate equation of $y(2)$ is given by:

$$f_{MRN-NA2}(y(2)) = k(4).\frac{K(2)^n}{y_{ss}(3)^n + K(2)^n} + k(9).\frac{K(5)}{y(2)+K(5)} - k(5).y(2) \tag{S8}$$

where

$$y_{ss}(3) = [\{k(6).\frac{K(1)}{y(1)+K(1)} + k(7).\frac{K(2)^n}{y(2)^n + K(2)^n} - k(5).K(5)\}$$

$$+ \{(k(6).\frac{K(1)}{y(1)+K(1)} + k(7).\frac{K(2)^n}{y(2)^n + K(2)^n} - k(5).K(5))^2$$

$$+ 4.k(5).K(5).(k(9) + k(6).\frac{K(1)}{y\ (1)+K(1)}$$

$$+ k(7).\frac{K(2)^n}{y(2)^n + K(2)^n})\}^{1/2}].\frac{1}{2.k(5)} \quad \text{(S9)}$$

and the noise function is expressed as [21, 45, 66]:

$$g_{MRN-NA2}(y(2)) = k(4).\frac{K(2)^n}{y_{ss}(3)^n + K(2)^n} + k(9).\frac{K(5)}{y(2)+K(5)} + k(5).y(2) \quad \text{(S10)}$$

In the same manner, the rate equation of $y(3)$ is given by:

$$f_{MRN-NA3}(y(3)) = k(6) + k(7).\frac{K(4)^n}{y_{ss}(2)^n + K(4)^n} + k(9).\frac{K(5)}{y(3)+K(5)} - k(8).y(3) \quad \text{(S11)}$$

where

$$y_{ss}(2) = [\{k(6).\frac{y(1)}{y(1)+K(1)} + k(4).\frac{K(2)^n}{y(3)^n + K(2)^n} - k(5).K(5)\}$$

$$+ \{(k(6).\frac{y(1)}{y(1)+K(1)} + k(4).\frac{K(2)^n}{y(3)^n + K(2)^n} - k(5).K(5))^2$$

$$+ 4.k(5).K(5).(k(9) + k(6).\frac{y(1)}{y(1)+K(1)}$$

$$+ k(4).\frac{K(2)^n}{y(3)^n + K(2)^n})\}^{1/2}].\frac{1}{2.k(5)}$$

(S12)

and the noise function is given by [21, 66]:

$$g_{MRN-NA}(y(3)) = k(6) + k(7).\frac{K(4)^n}{y_{ss}(2)^n + K(4)^n} + k(9).\frac{K(5)}{y(3)+K(5)} + k(8).y(3) \quad \text{(S13)}$$

Text S3

Stochastic potential profile analysis

We estimated the stochastic potential profile of the one-variable rate equation derived for the MRN and MRN-NA models as described in the Appendix F of reference [12].

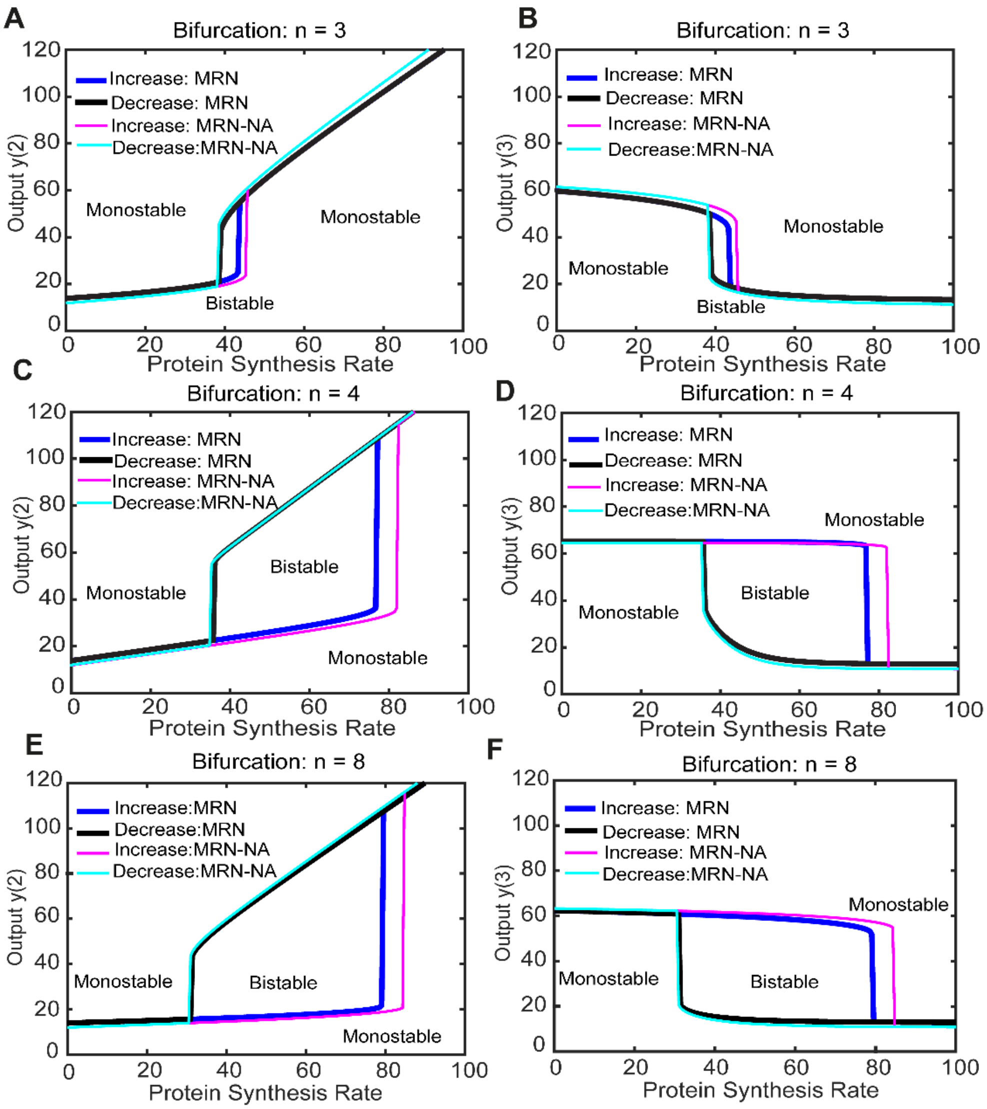

Figure S1: Bifurcation analysis of the MRN model and MRN-NA models

The steady-state trajectories of $y(2)$ (**A, C, E**) and of $y(3)$ (**B, D, F**) in the MRN and MRN-NA models are drawn with respect to protein synthesis rate constant $k(4)$. **A, B** for $n=3$; **C, D** for $n=4$; **E, F** for $n=8$. The parameters are given as $S=0$, $k(1)=100$, $k(2)=1$, $K(1)=K(3)=9$, $K(2)=K(4)=30$, $k(3)=k(6)=18.1$, $k(7)=43.1$, $k(5)=k(8)=0.8$, for the MRN model. The parameters are given as $S=0$, $k(1)=100$, $k(2)=1$, $k(4)=61.23$, $K(1)=K(3)=9$, $K(2)=K(4)=30$, $K(5)=K(6)=9$, $k(3)=k(6)=18.1$, $k(9)=k(10)=4.1$, $k(7)=43.1$, $k(5)=k(8)=0.8$, for the MRN-NA model.

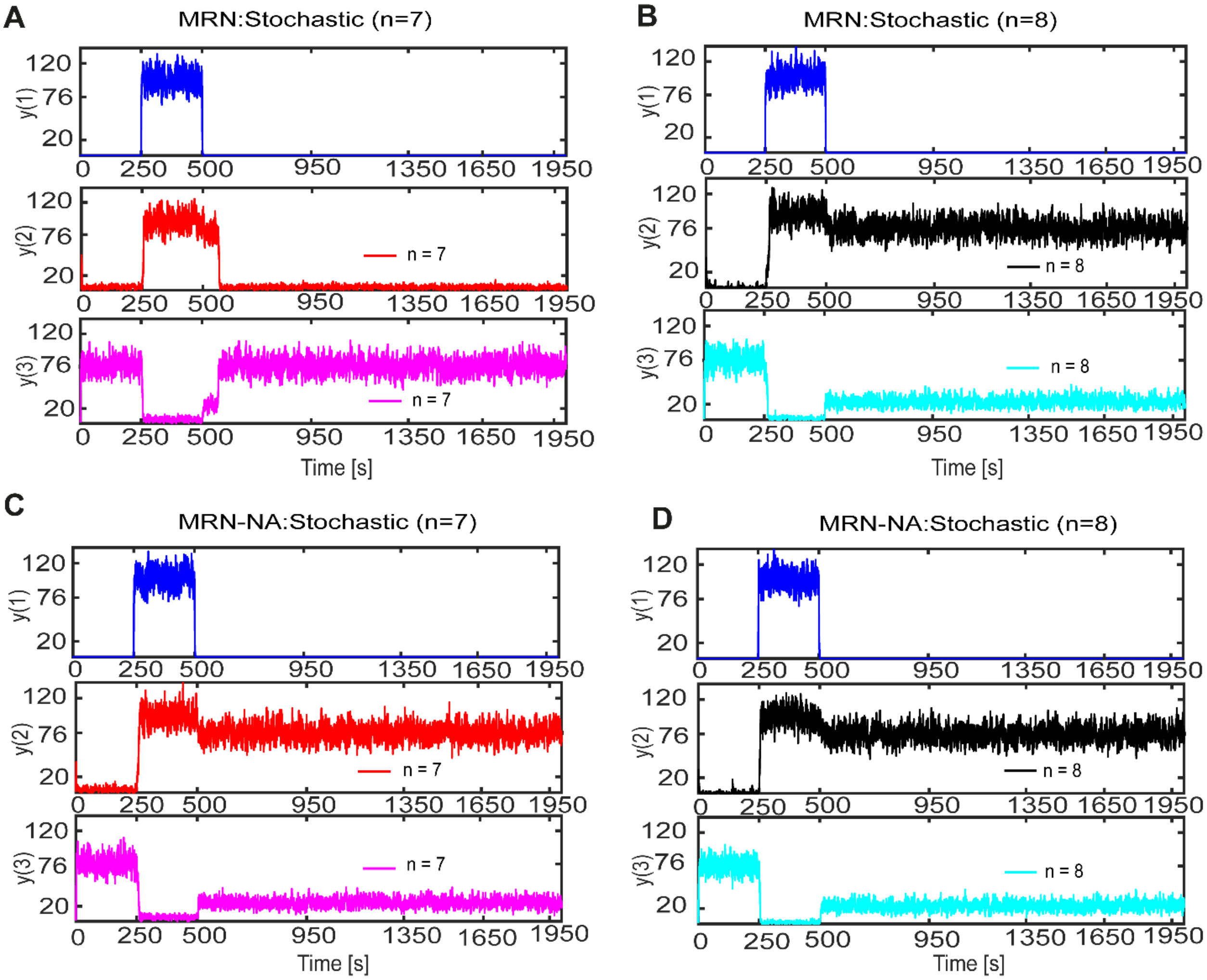

Figure S2: Stochastic simulations for 2000 simulation steps

A, B Trajectories of the stochastic simulation of $y(1)$, $y(2)$ and $y(3)$ at Hill coefficient $n=7$ and $n=8$ respectively for MRN model. The parameters are used as same as Fig 2. **C, D** Trajectories of the stochastic simulation of $y(1)$, $y(2)$ and $y(3)$ at Hill coefficient $n=7$ and $n=8$ respectively for MRN-NA model. The model parameters are the same as the ones used for Fig 3.

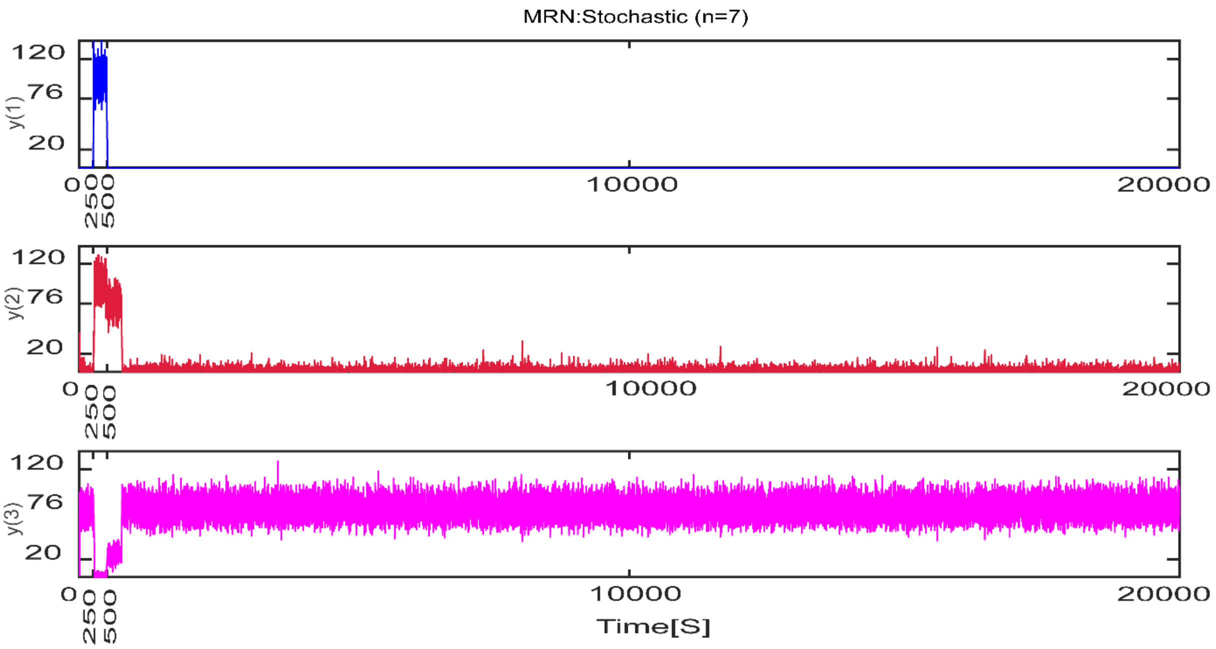

Figure S3: Stochastic simulation of the MRN for 20000 simulation time steps

Trajectories of the stochastic simulation of $y(1)$, $y(2)$ and $y(3)$ at Hill coefficient $n = 7$ for the MRN model. The parameters are the same as used for Fig 2.

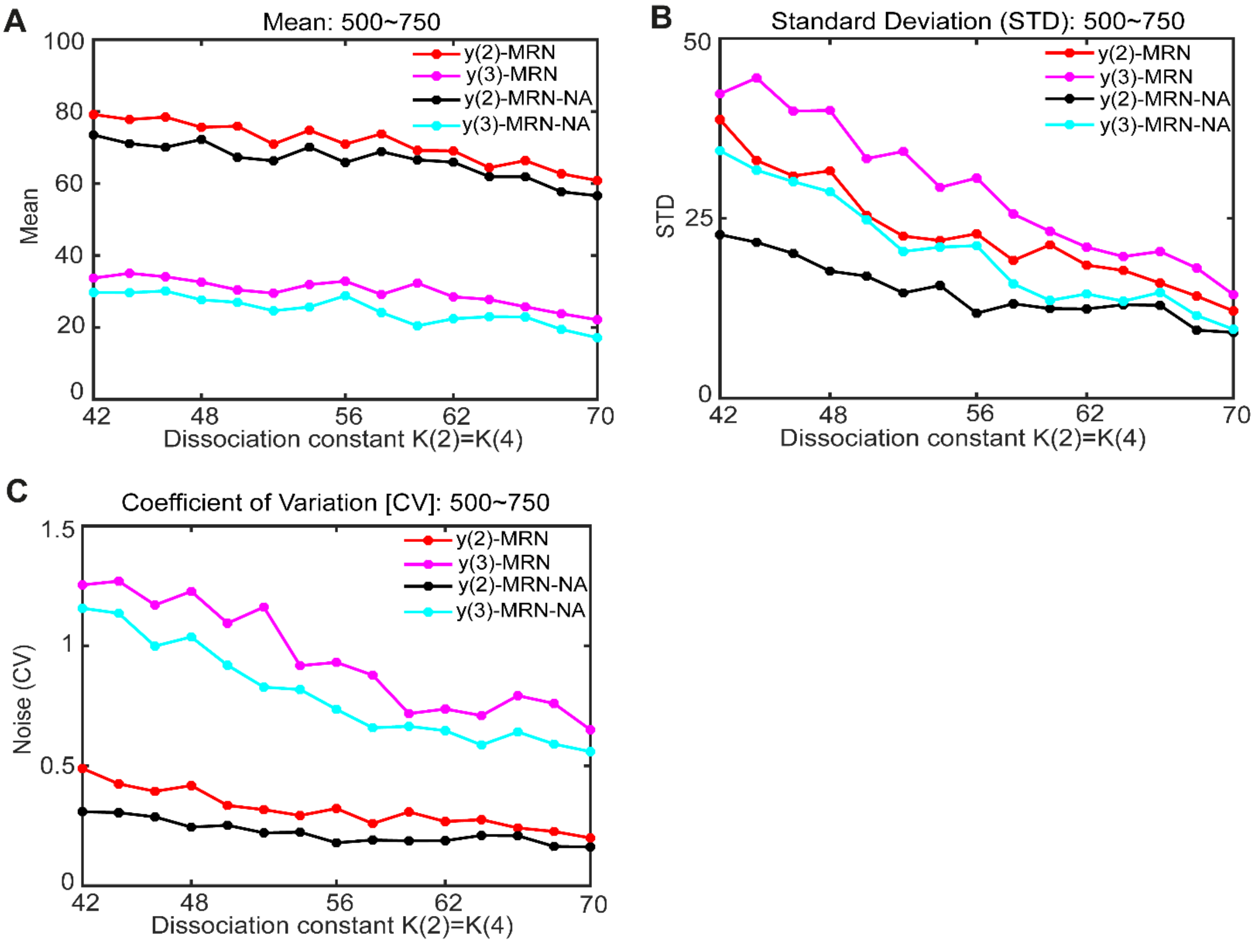

Figure S4: Stochastic fluctuations after the signal period

A, B, C Stochastic fluctuations in the MRN and MRN-NA models during the interval from time step 500 to 750. **A** Mean, **B** Standard deviation (SD), and **C** the coefficients of variation (CVs) are computed from the simulated stochastic trajectories after the signal period as a function of changing dissociation constants $K(2)=K(4)$ at Hill coefficient $n=8$. For more optimal comparability between the two models, the parameters associated to the negative autoregulation reactions were tuned to conserve the high steady state levels between both models. Shown are the Mean, SD and CVs of $y(2)$ (MRN: red line; MRN-NA: black line), and $y(3)$ (MRN: magenta line; MRN-NA: cyan line).

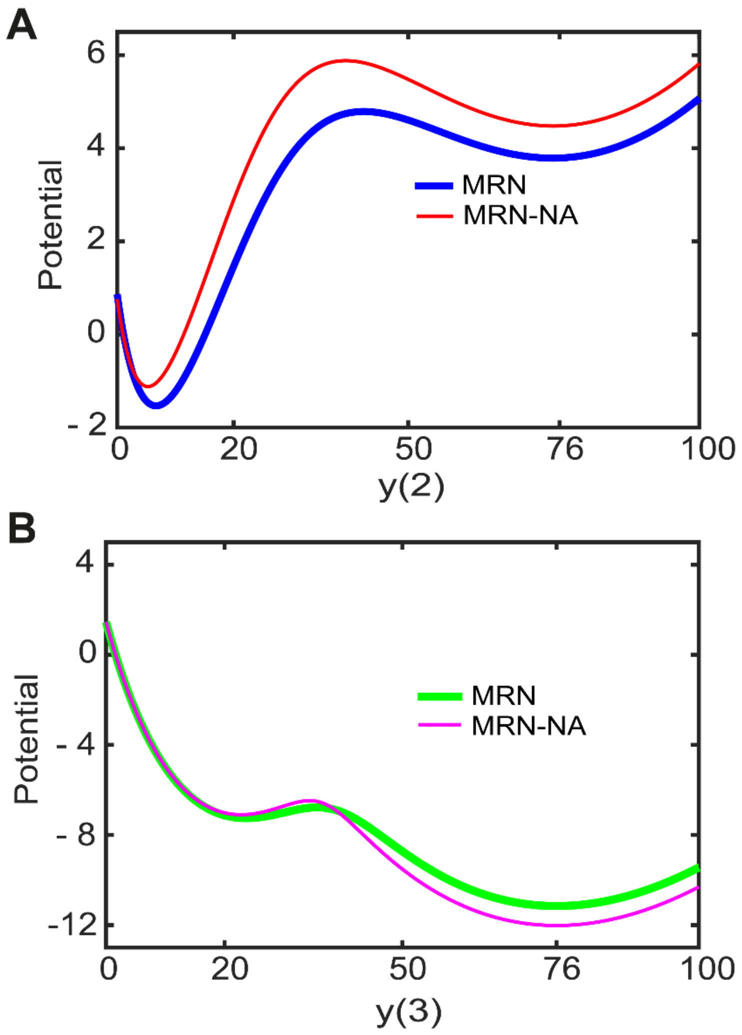

Figure S5: Stochastic potential profile of the MRN and MRN-NA models

A, B Double-well potential of **A** $y(2)$ and of **B** $y(3)$ in the MRN and MRN-NA models. The parameters are given as $S=0$, $k(1)=100$, $k(2)=1$, $K(1)=K(3)=9$, $K(2)=K(4)=30$, $k(3)=k(6)=18.1$, $k(4)=85.30>$ $k(7)=43.1$, $k(5)=k(8)=0.8$, $n=4$, for the MRN model. The parameters are given as $S=0$, $k(1)=100$, $k(2)=1$, $K(1)=K(3)=9$, $K(2)=K(4)=30$, $K(5)=K(6)=9$, $k(3)=k(6)=18.1$, $k(9)=k(10)=4.1$, $k(4)=90.65>$ $k(7)=43.1$, $k(5)=k(8)=0.8$, $n=4$, for the MRN-NA model.
